# Dysregulated Serum IL-23 and SIRT1 Activity in Peripheral Blood Mononuclear Cells of Patients with Rheumatoid Arthritis

**DOI:** 10.1371/journal.pone.0119981

**Published:** 2015-03-23

**Authors:** Daniel Wendling, Wasim Abbas, Marie Godfrin-Valnet, Amit Kumar, Xavier Guillot, Kashif Aziz Khan, Claire Vidon, Laurie Coquard, Eric Toussirot, Clément Prati, Georges Herbein

**Affiliations:** 1 Department of Rheumatology, Centre Hospitalier Régional Universitaire, Besançon, France; 2 Pathogens & Inflammation Laboratory, University of Franche-Comté, Besançon, France; 3 Department of Virology, Centre Hospitalier Régional Universitaire, Besançon, France; 4 Clinical Investigation Biotherapy Center506, Centre Hospitalier Régional Universitaire, Besançon, France; 5 Department of Therapeutics, University of Franche Comté, Besançon, France; 6 Structure Fédérative de Recherche 4234, University of Franche-Comté, Besançon, France; VU University Medical Center, NETHERLANDS

## Abstract

Sirtuin 1 (Sirt1) is a class III histone deacetylase (HDAC) that modulates gene expression and is involved in the regulation of proinflammatory cytokines. Interleukin-23 (IL-23) is produced by activated macrophages and dendritic cells and could fuel the progression of rheumatoid arthritis (RA). The goal of our study was to evaluate serum IL-23 levels and both Sirt1 activity and expression in peripheral blood mononuclear cells (PBMCs) in patients with RA compared to healthy controls (HC) and to determine the relationship between Sirt1 activity/expression and IL-23 levels. We assessed apoptosis in PBMCs of RA patients and its association with Sirt1 expression and serum IL-23. Serum IL-23 levels were increased in RA patients in comparison with controls. We found a positive correlation between the levels of serum IL-23 and serum IL-6 in RA patients. Decreased cytoplasmic Sirt1 activity was observed in RA patients with severe disease compared to HC. The expression of Sirt1 protein was significantly decreased in PBMCs of RA patients compared to HC using western blotting. Serum IL-23 levels correlated positively with the cytoplasmic Sirt1 activity in RA patients. Apoptosis rate of PBMCs isolated from RA patients was increased compared to HC and correlated negatively with the expression of Sirt1 protein and serum IL-23 levels. Levels of serum IL-23 and Sirt1 activity and expression were disturbed in RA parallel to increased PBMC apoptosis. Our findings might provide the rationale for the development of new therapeutic approaches in RA.

## Introduction

Rheumatoid arthritis (RA) is an inflammatory rheumatic disease characterized by chronic inflammation located on the peripheral joints [1]. Active pro-inflammatory cytokines such as tumor necrosis factor alpha (TNF), interleukin (IL)-1 beta or IL-6 are key players in the synovial inflammation of RA. Recently, the IL-6 related cytokine IL-23 has been reported to display proinflammatory properties [2,3]. Innate immune cells such as dendritic cells or monocytes/macrophages produce IL-23 [2,4–6]. IL-23 is involved in the development of Th17 response by the main cell type producing IL-17, the type 17 T helper cells (Th17) [4]. IL-17 is present at sites of inflammation and favors synovial inflammation in RA [4]. Additionally, IL-17 blockade limits inflammation and joint erosion [4]. The regulation of proinflammatory cytokines relies on epigenetic modifications that can modulate gene transcription and thus could regulate the production of proinflammatory cytokines [7]. The epigenetic control refers to several biochemical reactions on the DNA without changing its sequence, including histone modifications [8,9]. Methylation, ubiquitination, phosphorylation, sumoylation and acetylation can modify histone proteins. Histone acetylation is catalyzed by histone acetyl transferase (HAT) while deacetylation is influenced by histone deacetylase (HDAC). Based on the sequence homology with yeast, mammalian HDACs have been classified into four groups. Class I HDACs comprises of four members (HDAC 1, 2, 3 and 8). Class II is further divided into two subgroups and has total six members (HDAC 4,5,6,7,9,10). Class III HDACs comprises of seven sirtuins which require nicotinamide adenine dinucleotide (NAD+) for their enzymatic activity. Class IV has only one member (HDAC 11). Sirtuin 1 (Sirt1) is the most studied of the sirtuins with p53, nuclear factor-kappa B (NF-kappaB) and peroxisome proliferator-activated receptor-gamma coactivator (PGC)-1 alpha among others as targets. Sirt1 modulates the expression of genes involved in the regulation of various biological processes (cell survival, apoptosis, gluconeogenesis, adipogenesis, lipolysis), and local and systemic inflammation, as well as in bone and cartilage remodeling [10,11]. A ‘protective’ role of Sirt1 can be blocked by proinflammatory cytokines such as TNF, leading to inactivation of Sirt1 [12]. Sirt1 may be activated by several compounds [13], including resveratrol [14] and the homeostasis of Sirt1 expression has been reported to favor a long lasting healthy life [15].

The link between Sirt1 expression and activity and the control of proinflammatory cytokines such as IL-23 thus could be considered as a relevant marker for a sustained control of the inflammation process in several diseases including RA. There are limited results on the role of Sirt1 activity and expression and IL-23 production in patients with RA. In this study, therefore we aimed to evaluate both Sirt1 activity and expression in the peripheral blood mononuclear cells (PBMCs) of patients with RA and to analyze the potential association of serum IL-23 levels with Sirt1 activity and spontaneous apoptosis of PBMCs.

## Materials and Methods

All the patients and healthy controls (HC) gave their written informed consent to participate in the study according to the Helsinki declaration. The study protocol was approved by the local ethics committee (*Comité de Protection des Personnes EST-II*, *study registered under the number P/2009/85*).

### Patients

We enrolled 27 patients with RA meeting the 1987 American College of Rheumatology criteria. They were all receiving follow-up at the department of Rheumatology in the University Hospital of Besançon, France. Exclusion criteria were the presence of significant comorbidity (diabetes, dyslipidemia). In RA, disease activity and functional impairment were evaluated using the DAS28 score and Health Assessment Questionnaire (HAQ), respectively. Based on DAS28 score, most of the RA patients (60%) had a moderate disease activity (3.2≤DAS28≤5.1), 25% a high disease activity (DAS28>5.1) and 15% a low disease activity (DAS28<3.2). Erythrocyte sedimentation rate (ESR), C reactive protein (CRP) levels and circulating TNF, IL-6, and IL-8 were used as laboratory parameters to assess inflammation. Positivity for rheumatoid factors (68% of RA patients) and anti-CCP antibodies (76% of RA patients) were also recorded. In the RA group, patients were receiving low dosage corticosteroids (≤10 mg prednisone daily) and/or traditional DMARDs (methotrexate, leflunomide, sulfasalazine or hydroxychloroquine). At the time of inclusion, no RA patients were receiving TNF blocking agents.

### Control subjects

The control group consisted of 24 healthy subjects without inflammatory condition or treatment.

### Peripheral blood mononuclear cell isolation and cell lysate preparation

PBMCs were prepared from the peripheral blood of patients and HC and were cultured in RPMI-1640 medium supplemented with 10% (v/v) pooled AB human serum (Sigma), as previously described [16]. Isolation of nuclear and cytoplasmic extracts was performed as previously described [17]. Cells were washed with wash buffer (10 mM HEPES, pH 7.6, 10 mM KCl, 2 mM MgCl_2_, 1 mM EDTA). Cell pellets were then incubated on ice with cytoplasmic isolation buffer (10 mM HEPES, pH 7.6, 10 mM KCl, 2 mM MgCl_2_, 1 mM EDTA, 0.02% NP-40). Cytoplasmic extracts were collected by centrifugation and the nuclear pellets were washed twice in wash buffer, spun, and incubated for 15 min on ice with nuclear isolation buffer (20 mM HEPES pH 7.6, 420 mM NaCl, 1.5 mM MgCl_2_, 0.2 mM EDTA, 25% glycerol). Supernatants containing nuclear extracts were collected by centrifugation and stored at—80°C. Protease inhibitors (1 mM DTT, 1 mM PMSF, 1 microg/ml aprotinin, 1 microg/ml leupeptin, 1 microg/ml pepstatin) were added to all solutions. Protein concentration in nuclear and cytoplasmic extracts was determined by the Bradford method using a BioPhotometer (Eppendorf, Hamburg, Germany). The purity of cytoplasmic and nuclear extracts was further confirmed by the quantification of the expression of beta-actin, a cytoplasmic marker, and TATA-binding protein, a nuclear marker, using western-blotting (data not shown).

### Sirt1 activity and Sirt1 protein expression

Sirt1 activity was evaluated from cytoplasmic compartment using a fluorometric assay (SIRT1 fluorimetric kit, BML-AK-555, Enzo Life Sciences, Villeurbanne, France) at the 15 minutes point. Expression of Sirt1 protein was assessed using western blot as previously described [18]. Briefly, 10 microgram of cellular extracts were resolved on 10% SDS-PAGE using a Mini-PROTEAN 3 Cell (Bio-Rad Laboratories, Hercules, CA, USA). The proteins were electrotransferred onto a polyvinylidene difluoride membrane (Amersham Biosciences, Saclay, France) using Mini Trans-Blot Electrophoretic Transfer Cell (Bio-Rad Laboratories). The membranes were probed with primary antibodies followed by horseradish peroxidase-conjugated secondary immunoglobulin raised against the appropriate species; bands were detected using the ECL Plus kit (Amersham Biosciences). The primary antibodies used for western blot are as follows: rabbit anti-Sirt1 antibody (1:1000 dilution; Cell Signaling Technology, Beverly, MA) and mouse anti-beta-actin antibody (1:5000 dilution; Sigma-Aldrich, Saint-Louis, MO). Horseradish peroxidase-conjugated secondary antibodies goat anti-rabbit (1:5000 dilution; Santa Cruz Biotechnology, Santa Cruz, CA) and rabbit anti-mouse (1:5000 dilution; DakoCytomation, Trappes, France) were used.

### Measurement of IL-23, TNF, IL-6 and IL-8

Circulating IL-23, TNF, IL-6 and IL-8 were evaluated in sera from patients and HC using ELISA assays (Quantikine assay, R&D Systems, Minneapolis, MN). Sensitivity of the assays for TNF, IL-6, IL-23 and IL-8 detected a few pg/ml.

### Detection of apoptosis

The detection of apoptosis by annexin V assay (BD Pharmingen) was performed on PBMCs isolated from RA patients and HC cultured in serum-free medium for 48 hours as previously described [19]. Briefly, PBMCs were washed twice with ice cold phosphate buffered saline (PBS) and resuspended in 1X annexin binding buffer containing annexin V as per manufacturer’s instructions (BD Pharmingen). Cells were gently vortexed and incubated at room temperature for 15 minutes in dark. Cell suspension volume was made up to 500μl with 1X annexin V binding buffer and analyzed in flow cytometer (BD FACS Calibur) for annexin V staining.

### Statistical analysis

Results were expressed as mean ± standard deviation (SD). The student T-test was used for statistical analysis between the groups (RA and HC). Values of *p* less than 0.05 were considered significant. The Pearson correlation coefficient was used to measure the strength of a linear association between two variables.

## Results

### Increased serum IL-23 levels in RA patients

**Fig 1:**
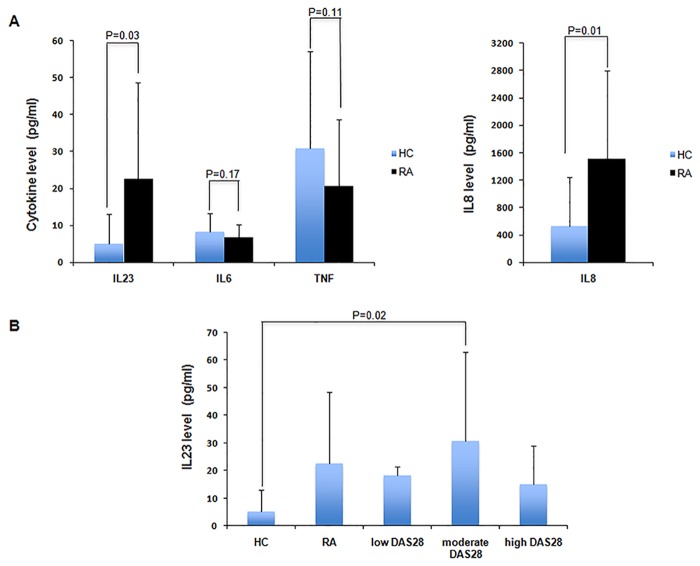
Quantification of levels of IL-23, IL-6, IL-8 and TNF in serum of HC and RA subjects. A. Levels of IL-23, IL-6, IL-8 and TNF in serum of healthy controls and patients with RA. B. Serum levels of IL-23 in subpopulations of RA patients based on DAS 28 score. IL-23, IL-6, IL-8 and TNF were measured by ELISA. Results were expressed as means ± SD. (HC: healthy controls; RA: rheumatoid arthritis).

**Fig 2:**
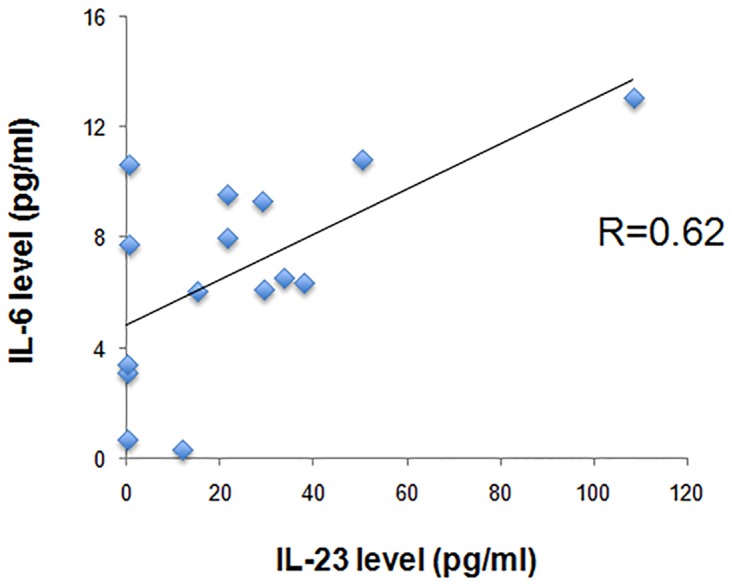
IL-23 levels correlated positively with IL-6 levels in serum of RA patients. Levels of IL-23 and IL-6 were measured in the serum of RA patients. IL-23 levels correlated positively with IL-6 levels in serum of RA patients (r = 0.62).

Patients with RA had mostly mild disease activity (DAS28 = 4.27±1.07). The biochemical parameters of inflammation investigated (CRP, ESR) were higher in patients with RA compared to HC (CRP p = 0.001; ESR p = 0.01) (Table 1). Serum IL-8 levels were 2.8-fold higher in RA patients compared to HC (p = 0.01) (Table 1, Fig. 1A). In contrast no significant difference was observed for the levels of IL-6 (p = 0.17) and TNF (p = 0.11) in RA patients compared to HC (Table 1, Fig. 1A). Serum IL-23 levels were 4.4-fold higher in RA compared to HC (mean values 22.7±25.9 pg/ml versus 5.1±7.9 pg/ml, P = 0.03) (Table 1, Fig. 1A). Additionally among RA patients, the highest serum IL-23 levels were observed in patients with mild disease and the lowest in patients with severe disease (Fig. 1B). Serum levels of IL-23 correlated positively with serum IL-6 levels (r = 0.62) (Fig. 2), but neither with TNF nor with IL-8 levels (r = 0.10 and r = 0.06, respectively) (data not shown).

**Table 1:** Biological data of the studied patients with rheumatoid arthritis and healthy controls.

Mean±SD	Age (years)	Sex ratio (M/F)	Sirt1 Cytopl. activity (AFU)	CRP mg/l	ESR mm/h	DSA28	IL-23 pg/ml	TNF pg/ml	IL-6 pg/ml	IL-8 pg/ml
**Control subjects N = 24**	54±13	1.12	356069± 194855	2.6± 1.6	8.6± 5.3	na	5.1± 7.9	30.9± 26.2	8.3± 4.9	527.4± 709.6
**RA patients N = 27**	55±12	0.47	283818± 179670	12.9± 12.3	21.4± 13.7	4.27± 1.07	22.7± 25.9	20.7± 18.1	6.8± 3.5	1503± 1297
P*	0.38	0.10	0.15	0.001	0.01	na	0.03	0.11	0.17	0.01

Abbreviations: AFU, Arbitrary fluorescence units; CRP, C-Reactive Protein; ESR, Erythrocyte sedimentation rate; IL, interleukin; TNF, Tumor necrosis factor; na, not available.

*P value <0.05: significant

### Serum IL-23 levels correlated positively with cytoplasmic Sirt1 activity in PBMCs isolated from RA patients

**Fig 3:**
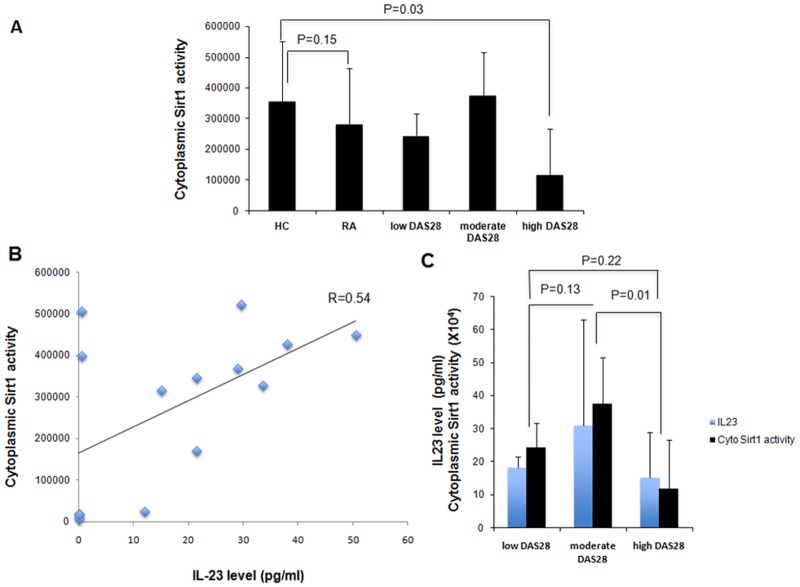
Serum IL-23 levels correlated positively with cytoplasmic Sirt1 activity in PBMCs isolated from RA patients. A. Cytoplasmic Sirt1 activity in PBMCs from subpopulations of RA patients based on DAS 28 score. Cytoplasmic Sirt1 activity was measured in PBMCs of subpopulations of RA patients based on DAS 28 score as described in Materials and Methods. Results were expressed as means ± SD. B. Cytoplasmic Sirt1 activity correlated positively with serum IL-23 levels (r = 0.54). C. The highest serum IL-23 levels and cytoplasmic Sirt1 expression were observed in RA with mild disease. Results were expressed as means ± SD.

**Fig 4:**
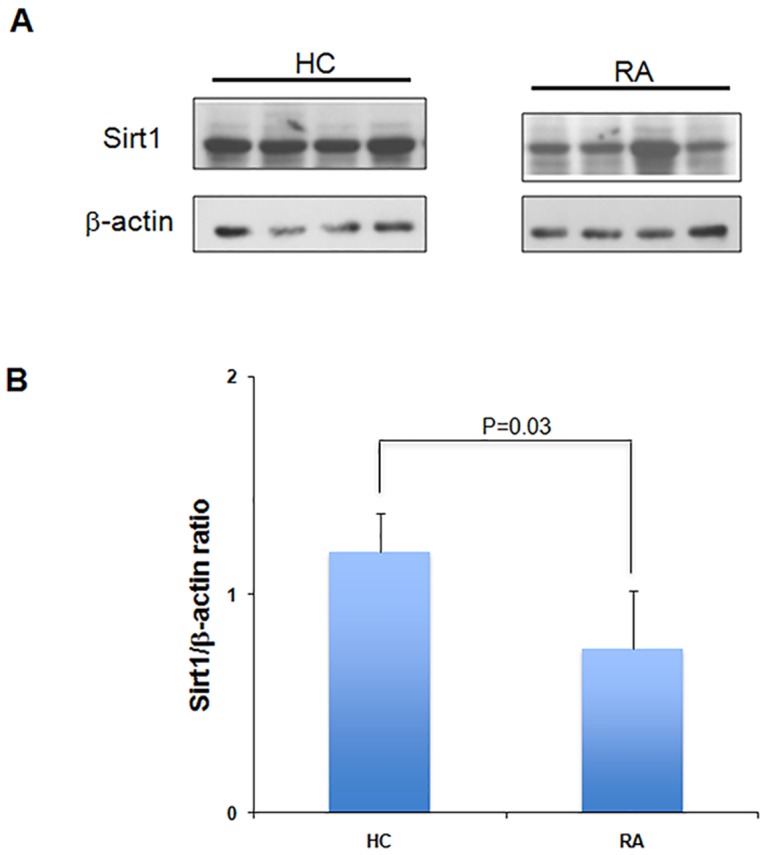
Expression of Sirt1 protein is reduced in PBMCs of RA patients compared to HC. A. Sirt1 protein expression was measured in PBMCs isolated from RA and HC using western blotting as described in Materials and Methods. Beta-actin was used as a loading control. B. Histogram represents Sirt1/beta-actin ratio in PBMCs of healthy controls and patients with RA. Protein levels of Sirt1 and beta-actin were quantified by densitometry using ImageJ 1.40 software. Results were expressed as means ± SD. P = 0.03

We did not find significant difference in the cytoplasmic Sirt1 activity in PBMCs of RA patients compared to HC (283818±179670 versus 356069±194855, p = 0.15) (Table 1) as reported previously [20]. Cytoplasmic Sirt1 activity was lower in RA patients with severe disease compared to HC (120063±147394 versus 356069±194855; P = 0.03) (Fig. 3A). Cytoplasmic Sirt1 activity correlated positively with serum IL-23 levels (r = 0.54) (Fig. 3B) and IL-6 levels (r = 0.65) (data not shown), but only very weak or absence of correlation was observed with TNF and IL-8 levels (r = 0.21 and r = -0.09, respectively). The highest serum IL-23 levels and cytoplasmic Sirt1 expression were observed in RA with mild disease (Fig. 3C). To determine whether decreased Sirt1 activity in RA resulted from decreased Sirt1 protein expression, we assessed Sirt1 protein expression in PBMCs isolated from RA and HC using western blotting. The Sirt1 protein expression was reduced by 1.6-fold in PBMCs of RA patients compared to HC (p = 0.03) (Fig. 4A & 4B).

### Serum IL-23 levels correlated negatively with apoptosis of PBMCs isolated from RA patients

**Fig 5:**
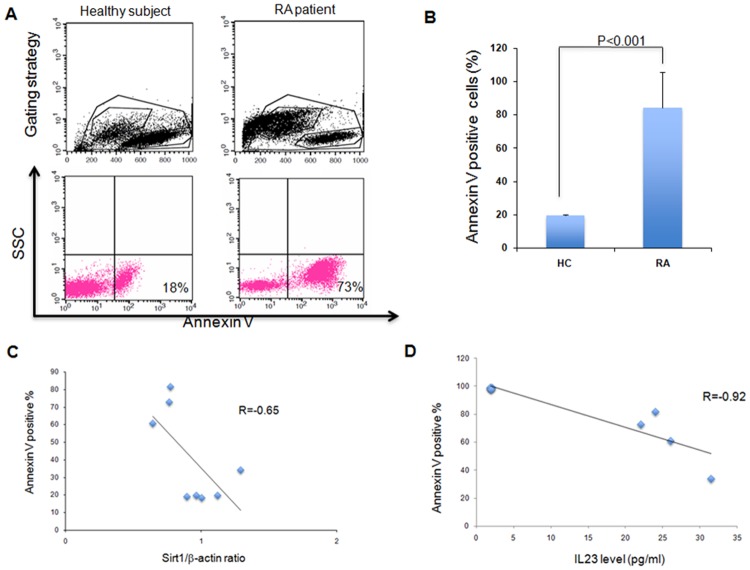
Serum IL-23 levels correlated negatively with spontaneous apoptosis of PBMCs isolated from RA patients. A. B. Spontaneous apoptosis of PBMCs from healthy controls and patients with RA after culture in RPMI without serum for 48 hours. Apoptosis was measured by annexin-V assay as reported in the Materials and Methods chapter. Flow cytometric data are representative of five independent experiments. Percentage of apoptotic cells is indicated. SSC, size scattered count. The histogram summarizes the spontaneous apoptosis of PBMCs from healthy controls and patients with RA after culture in RPMI without serum for 48 hours. The results represent means ± SD. P<0.001. C. Expression of Sirt1 protein correlated negatively with the rate of spontaneous apoptosis in PBMCs isolated from RA patients (r = -0.65). D. Serum levels of IL-23 correlated negatively with apoptosis of PBMCs isolated from RA patients (r = -0.92).

Since Sirt1 can inhibit apoptosis and promote cell survival in several cell types [21–23], apoptosis was then assessed in PBMCs of RA and HC using annexin-V assay. Rates of apoptosis were 4.3-fold higher in PBMCs of RA patients compared to controls (P<0.001) (Fig. 5A & 5B). Expression of Sirt1 protein correlated negatively with the rate of apoptosis in PBMCs with decreased expression of Sirt1 protein and high rate of apoptosis in PBMCs isolated from RA patients (r = -0.65) (Fig. 5C). Serum levels of IL-23 correlated negatively with apoptosis of PBMCs isolated from RA patients (r = -0.92) (Fig. 5D).

## Discussion

Our results indicate that serum IL-23 levels are increased in RA patients compared to HC (Fig. 1). Sirt1 activity was reduced in patients with severe RA (Fig. 3C) and Sirt1 expression was decreased in patients with RA (Fig. 4). We observed a positive correlation between serum IL-23 levels and Sirt1 activity in PBMCs of RA patients (Fig. 3C). Increased apoptosis was detected in PBMCs isolated from RA patients compared to HC. Expression of Sirt1 protein was decreased in PBMCs of RA patients suggesting a negative correlation between rate of apoptosis and Sirt1 expression (Figs. 4–5). Serum IL-23 levels correlated negatively with apoptosis in PBMCs isolated from RA patients. Altogether our results indicate imbalanced IL-23 production, decreased Sirt1 activity and expression in PBMCs from RA patients with increased PBMC apoptosis.

We have shown previously that the measurement of Sirt1 activity in PBMCs drawn from peripheral blood is feasible, allowing an accessible cell subset for investigation [20]. We also observed in our previous reports that Sirt1 activity is present not only in the nuclear compartment but also in the cytoplasmic one and both are correlated [20]. Therefore in the present study, we performed the measurement of Sirt1 activity solely in the cytoplasmic compartment. An impairment of Sirt1 activity was hypothesized in patients with RA, however no difference was found between patients with RA and controls in regard to cytoplasmic Sirt1 activity as reported previously [20]. Since Sirt1 activity is supposed to decrease with age, we matched the RA patients and HC for age (Table 1). Nevertheless, several other factors including diabetes and obesity have been reported to modulate Sirt1 activity [24]. We found that cytoplasmic Sirt1 activity was lower in RA with severe disease compared to HC, indicating that Sirt1 activity depends on the severity and/or the duration of the disease. Lower Sirt1 activity in RA patients with severe disease could be explained by immunosenescence [25,26]. In agreement with our observation, we reported recently that Sirt1 activity (cytoplasmic and nuclear) was lower in ACPA-positive RA compared to ACPA-negative RA [20].

Although the literature has previously shown mainly the nuclear activity of Sirt1 [14], at least two different mechanisms could account for its biological action. Sirt1 preferentially deacetylates lysine 9 of histone H3 and lysine 16 of histone H4 [27]. Additionally, Sirt1 interacts directly with the p65 subunit of NF-kappa B, leading to deacetylation at lysine 310, culminating in decreased NF-kappa B-associated transcription [28]. Sirt1 deacetylates lysine 310 of RelA/p65 without affecting the acetylation status of other lysine residues [28]. Following treatment with resveratrol, a Sirt1 activator, the localization of both Sirt1 and RelA/p65 proteins on the gene promoter suggests that Sirt1 may actively repress gene expression by deacetylating RelA/p65 directly on chromatin [27]. In the case of NF-kappa B, the heterodimer composed of RelA/p65 and p50 proteins interacts also with HDAC1, 2 and 3 enzymes, making the regulation of NF-kappa B-dependent genes even more complex [29]. Recently, the cathepsin B-mediated cleavage of Sirt1 by TNF has been reported [12]. Interestingly, other proinflammatory cytokines participating in arthritis, including IL-6, have been reported to activate cathepsin B and therefore could also trigger the cleavage of Sirt1 [30]. IL-23 stimulation has been reported to activate cathepsin K through up-regulation of expression of the receptor activator of NF-B ligand (RANKL) [31]. *In vitro* cleavage of recombinant Sirt1 has been reported with several members of cathepsin family including cathepsin B, L and S [32]. Since we observed that serum IL-23 levels correlated positively with cytoplasmic Sirt1 activity in PBMCs isolated from RA patients, we cannot exclude that cathepsins could be involved in the degradation of Sirt1 mediated by IL-23, thereby providing a negative feed-back loop.

We observed a positive correlation between serum IL-23 levels and Sirt1 activity in PBMCs isolated from RA patients. In agreement with our findings, recent reports indicate that Sirt1 can stimulate proinflammatory cytokine production [23,33]. Additionally, Sirt1 is a key regulator of the interleukin-12 p70/IL-23 balance in human dendritic cells [34]. HDAC inhibitors inhibit IL-6 release by bone marrow-derived macrophages exposed to microbial products such as lipopolysaccharides (LPS) and heat-killed *Escherichia coli* and *Staphylococcus aureus* [35,36] and decrease both IL-6 and TNF production in PBMCs isolated from RA patients [19,37]. Givinostat (originally called ITF2357) is a hydroxamic acid which inhibits class I and class II HDACs and reduces the production and release of proinflammatory cytokines including TNF, IL-1beta and IL-6 from human blood monocytes [38]. HDAC inhibitors suppress inflammatory activation of RA [39,40]. Sirtuin inhibition decreases the production of TNF, IL-6 and RANTES (regulated upon activation normal T cell expressed and secreted) in LPS-stimulated macrophages [33], suggesting that sirtuin activation may favor proinflammatory cytokine production by activated macrophages.

In agreement with this hypothesis, inhibition of Sirt1 enzymatic activity reduces LPS-induced levels of TNF in monocytes of patients with rheumatoid arthritis [23]. We also observed a positive correlation between IL-23 and IL-6 levels in serum of RA patients. IL-23 and IL-6 are two closely related proinflammatory cytokines that are both produced by activated macrophages [4,41,42]. By contrast, other studies indicate that resveratrol inhibits TNF-induced inflammation via Sirt1 [43], and suppresses expression of TNF, IL-6 and IL-8 [44]. Sirt1 deacetylates the p65 subunit of NF-kappaB at lysine 310, attenuating NF-kappa B transcriptional activation, and thereby could decrease proinflammatory cytokine production. Altogether, Sirt1 activity and subsequent proinflammatory cytokine production such as IL-23 may depend on the cell type involved (monocytes or macrophages, or PBMCs in peripheral blood compartment versus cells of the cartilage tissue) and on the activation state of the cell type studied. Recently, a subpopulation of monocytes namely CD56+ monocytes, have been reported to have a dysregulated cytokine response to LPS with enhanced IL-23 production and to accumulate in RA and immunosenescence [25]. Increased plasma IL-23 levels are associated with increased disease activity in patients with early RA [45] and systemic levels of IL-23 are strongly associated with disease activity in RA [46]. We found a peak of serum IL-23 levels in RA patients with mild disease and lower serum IL-23 levels in early and late disease. Future studies will assess whether the lower serum levels of IL-23 observed in severe RA could be explained by some defects in antigen-presenting cells [4, 41]. Sirt1 activity declines with age, potentially due to ROS-mediated depletion of NAD^+^, and a compensatory mechanism exists to increase Sirt1 protein levels to increase the relative success of Sirt1 when competing for the limited NAD^+^ pool [47]. Additionally IL-23 production is increased during ageing in murine models [48]. Aging dependent upregulation of IL-23p19 gene expression in dendritic cells is associated with differential transcription factor binding and histone modifications [48]. Sirt1 could play a role in IL-23p19 gene expression by modulating histone acetylation. Elevated synthesis and secretion of IL-23 in dendritic cells from aged mice could be one of the possible mechanisms by which T cell maturation is shifted towards a Th17 phenotype with age, increasing autoimmune conditions in the elderly [49]. New treatments such as blocking Th17-polarizing cytokines by HDAC inhibitors could ultimately decrease the production of IL-23/IL-17 cytokines [50,51].

It has been demonstrated that Sirt1 regulates apoptosis- and cartilage-specific gene expression in human chondrocytes and mouse models [52,53]. Mice without Sirt1 activity are characterized by reduced levels of type II collagen, aggrecan, and glycosaminoglycan, elevated levels of matrix metalloproteinases 8, 9 and 13 in the cartilage, and elevated apoptosis of chondrocytes. Sirt1 overexpression in RA synovial fibroblasts protected cells from apoptosis parallel to the production of proinflammatory cytokines IL-6 and IL-8 [23]. In agreement with previous findings [23, 54], we found that expression of Sirt1 protein correlated negatively with the rate of apoptosis in PBMCs isolated from RA patients. Although IL-23 induces apoptosis of self-reactive thymocytes in thymic negative selection [55], we observed that serum levels of IL-23 correlated negatively with apoptosis of PBMCs isolated from RA patients, indicating a possible link between the production of IL-23 by monocytes/macrophages and resistance to apoptosis of PBMCs. Spontaneous apoptosis of CD4+CD25+ T cells from active RA patients has found to be increased in comparison with controls [56]. By contrast, deficient *in vitro* spontaneous apoptosis of monocytes isolated from RA patients has also been reported [57]. Therefore spontaneous apoptosis in PBMCs isolated from RA patients and its regulation by IL-23 might depend on the stage of the disease and on the cell type involved (monocytes versus peripheral blood lymphocytes).

In conclusion, we observed dysregulated IL-23 production, decreased SIRT1 activity and expression in PBMCs from RA patients with increased PBMC apoptosis. The molecular mechanisms underlying IL-23 production, Sirt1 activity and apoptosis in PBMCs of RA patients are complex, and a better understanding of this interplay may lead to new therapeutic approaches.
